# Circulating serum xenoestrogens and mammographic breast density

**DOI:** 10.1186/bcr3432

**Published:** 2013-05-27

**Authors:** Brian L Sprague, Amy Trentham-Dietz, Curtis J Hedman, Jue Wang, Jocelyn DC Hemming, John M Hampton, Diana SM Buist, Erin J Aiello Bowles, Gale S Sisney, Elizabeth S Burnside

**Affiliations:** 1 Department of Surgery, University of Vermont, Fletcher House 301, 111 Colchester Ave, Burlington, VT 05401, USA; 2 Department of Population Health Sciences, University of Wisconsin, 610 Walnut St, Madison, WI 53726, USA; 3 University of Wisconsin Carbone Cancer Center, 600 Highland Ave, Madison, WI 53792, USA; 4 Environmental Health Division, Wisconsin State Laboratory of Hygiene, 465 Henry Mall, Madison, WI 53706, USA; 5 Group Health Research Institute, 1730 Minor Ave 1600, Seattle, WA 98101, USA; 6 Department of Radiology, University of Wisconsin, E3/366 Clinical Science Center, Madison, WI 53792, USA

**Keywords:** mammographic density, breast cancer, endocrine disruptors, epidemiology

## Abstract

**Introduction:**

Humans are widely exposed to estrogenically active phthalates, parabens, and phenols, raising concerns about potential effects on breast tissue and breast cancer risk. We sought to determine the association of circulating serum levels of these chemicals (reflecting recent exposure) with mammographic breast density (a marker of breast cancer risk).

**Methods:**

We recruited postmenopausal women aged 55 to 70 years from mammography clinics in Madison, Wisconsin (*N *= 264). Subjects completed a questionnaire and provided a blood sample that was analyzed for mono-ethyl phthalate, mono-butyl phthalate, mono-benzyl phthalate, butyl paraben, propyl paraben, octylphenol, nonylphenol, and bisphenol A (BPA). Percentage breast density was measured from mammograms by using a computer-assisted thresholding method.

**Results:**

Serum BPA was positively associated with mammographic breast density after adjusting for age, body mass index, and other potentially confounding factors. Mean percentage density was 12.6% (95% confidence interval (CI), 11.4 to 14.0) among the 193 women with nondetectable BPA levels, 13.7% (95% CI, 10.7 to 17.1) among the 35 women with detectable levels below the median (<0.55 ng/ml), and 17.6% (95% CI, 14.1 to 21.5) among the 34 women with detectable levels above the median (>0.55 ng/ml; *P*_trend _= 0.01). Percentage breast density was also elevated (18.2%; 95% CI, 13.4 to 23.7) among the 18 women with serum mono-ethyl phthalate above the median detected level (>3.77 ng/ml) compared with women with nondetectable BPA levels (13.1%; 95% CI, 11.9 to 14.3; *P*_trend _= 0.07). No other chemicals demonstrated associations with percentage breast density.

**Conclusions:**

Postmenopausal women with high serum levels of BPA and mono-ethyl phthalate had elevated breast density. Further investigation of the impact of BPA and mono-ethyl phthalate on breast cancer risk by using repeated serum measurements or other markers of xenoestrogen exposure are needed.

## Introduction

Humans are widely exposed to estrogenically active environmental chemicals ("xenoestrogens") in the course of everyday life. Phthalates, parabens, and phenols are three of the most common classes of xenoestrogens found in foods and consumer products. Phthalates are used as a plasticizer in many consumer plastics, adhesives, detergents, and pharmaceuticals, and are also found in personal care products, such as shampoos, lotions, and shaving products [1]. Parabens are used as a preservative in many of the same personal care products and pharmaceuticals, and are additionally used as antimicrobials in foods [2]. Phenols are commonly used in the manufacture of consumer products made of polycarbonate plastics, the coatings of food containers, and as surfactants in detergents and personal care products [3]. Data from the National Health and Nutrition Examination Survey (NHANES) show that the most common phthalates, parabens, and phenols are detectable in the urine of more than 90% of Americans [4-6].

Health concerns regarding exposure to xenoestrogens stem from their potential actions as endocrine disruptors. Laboratory studies have demonstrated that many phthalates, parabens, and phenols can bind to and activate the estrogen receptor, promote the proliferation of human breast cancer cells, or increase uterine weight in immature mice [2,7-9]. Many of these chemicals have the ability to induce additional biologic effects, including DNA damage, altered DNA methylation, and altered sex hormone metabolism [10-13].

Investigation of the health effects of these chemicals in humans is limited by challenges in exposure assessment because of their rapid metabolism. Measurements of phthalates, parabens, and phenols in urine and serum primarily reflect exposures within the past 24 hours [1-3], which may not be correlated with long-term exposure. However, a handful of studies have reported associations with various health outcomes. Elevated serum bisphenol A (BPA) levels were associated with recurrent miscarriage in a small case-control study [14], and elevated urinary BPA levels were associated with cardiovascular disease in NHANES [15]. A variety of studies have reported links between urinary or serum phthalate levels and impaired sperm function in men [16], endometriosis in women [17], early puberty [18], and premature breast development [19]. Most recently, a case-control study of women in Northern Mexico found that urinary levels of mono-ethyl phthalate were positively associated with breast cancer risk [20]. These findings raise important questions regarding the potential impacts of phthalates and other similar chemicals on breast tissue.

Mammographic breast density is one of the strongest risk factors for breast cancer, and a useful marker for the effects of various exposures on breast tissue [21]. Mammographic breast density refers to the appearance of breast tissue on a mammogram, reflecting the relative amounts of radiodense epithelial and stromal tissue versus radiolucent fat tissue; breast density is qualitatively rated by radiologists from mammograms on a routine basis but may also be measured quantitatively for research through various techniques [21]. A meta-analysis has estimated that women with mammographically dense tissue in 75% or more of the total breast area have a 4.2-fold increase in breast cancer risk compared with women with less than 5% mammographic breast density [22]. Numerous breast cancer risk factors have been associated with mammographic breast density [21], and mammographic breast density responds to changes in exposures, including postmenopausal hormone use [23,24] and chemoprevention with tamoxifen [25].

We hypothesized that circulating serum levels of phthalates, parabens, and phenols may be positively associated with mammographic breast density. We examined this relation in the Wisconsin Breast Density Study, a cross-sectional study of postmenopausal women receiving a screening mammogram.

## Materials and methods

### Study population

The Wisconsin Breast Density Study is a cross-sectional study of women receiving screening mammograms at the UW Health West Clinic or UW Health Breast Center in Madison, Wisconsin [26,27]. The study was approved by the University of Wisconsin Health Sciences Institutional Review Board, and all subjects provide written informed consent. Details on subject recruitment were previously described [27]. In brief, eligibility was limited to postmenopausal women between the ages of 55 and 70 years who attended the mammography clinics for a screening mammogram between June 2008 and June 2009. Eligibility was further limited to women with no history of postmenopausal hormone use, breast implants, or a previous diagnosis of breast cancer. In total, 268 subjects were enrolled in the study.

### Data collection

Each subject completed a study questionnaire and provided a blood sample immediately after completion of the screening mammogram. The questionnaire assessed established breast cancer risk factors and known correlates of mammographic breast density, including demographic and anthropometric factors, reproductive and menstrual history, family history of breast cancer, and lifestyle factors such as alcohol consumption, smoking, and physical activity.

A 30-ml blood sample was collected from each subject by venipuncture into uncoated glass vacutainer tubes (Fisher Scientific, Pittsburgh, PA, USA). Immediately after spinning down of the sample, 4.5 ml of serum was transferred into borosilicate glass vials (Wheaton Science Products, Millville, NJ, USA). The glass vials were prepared by baking at 450°C to burn off all organic carbon, and the Teflon-coated caps were sonicated in methanol to remove any contaminants. The caps and vials were then assembled in a biosafety cabinet and remained sealed until the serum sample was collected. The serum samples were stored frozen in the glass vials at -70°C until thawed for analysis.

Free concentrations of phthalates, parabens, and phenols were quantified at the Wisconsin State Laboratory of Hygiene by using methods based on solid-phase extraction (Strata-X; Phemomenex, Torrance, CA, USA) and isotope dilution high-performance liquid chromatography (Agilent 1100, Waldbronn, Germany) with tandem mass spectrometry (API4000; AB/SCIEX, Framingham, MA, USA) with APCI negative ionization [28,29]. Analytical quality assurance (QA) parameters included reagent (all <LOD) and method blanks (all <LOD with exception of nonylphenol, for which five of nine were >LOD), calibration check standards (recovery = 98.7% to 114.1%; *n *= 31 for phthalates and parabens and *n *= 20 for phenols), and double charcoal-treated human serum matrix control spikes at low (1 ng/ml; recovery = 82.9% to 114%; *n *= 12 for phthalates and parabens and *n *= 14 for phenols) and mid (5 and 10 ng/ml; recovery = 87.4% to 112.9%; *n *= 12 for phthalates and parabens and *n *= 19 for phenols) calibration-curve levels. Lower limits of detection were based on observed 3:1 signal-to-noise ratios, and are listed in Table 2.

As previously described [27], endogenous estradiol was measured at the Reproductive Endocrine Research Laboratory at the University of Southern California by using validated radioimmunoassays.

Mammographic breast density was quantitatively assessed as previously described [26,27]. All subjects received a screening mammogram on a digital machine. Full-resolution digital images of the craniocaudal view of the left breast were analyzed for breast density by using a computer-assisted thresholding technique implemented by Cumulus software [30]. Total breast area and dense area were recorded by a single trained operator (EJAB) with high reliability (intraclass correlation coefficients >0.92 for repeated measures). Percentage breast density was calculated as the ratio of dense to total breast area. All enrolled subjects were included in the study, regardless of the clinical assessment of their mammograms.

### Statistical analysis

All statistical analyses were performed by using SAS Statistical Software (Version 9.2; SAS Institute, Inc., Cary, NC, USA). Insufficient serum was available for four study subjects, leaving a total of 264 samples for analysis. Serum propyl paraben level was missing for one woman, serum octylphenol was missing for one woman, body mass index was missing for two women, and education level was missing for one woman. Multiple imputation was used for missing covariate data, with 10 imputations using the Markov Chain Monte Carlo method [31]. The imputation model contained percentage breast density and all variables listed in Tables 1 and 2. For statistical analyses, each model was fit separately to the 10 imputed datasets, and the results combined for statistical inferences by using the methods of Rubin [32].

**Table 1:** Characteristics of study participants (*N *= 264), Wisconsin Breast Density Study, 2008 to 2009

	Mean ± SD or *n *(%)
Age (years)	60.6 ± 4.4
Body mass index (kg/m^2^)^a^	28.9 ± 6.6
First-degree family history of breast cancer	63 (23.9)
Nulliparous	67 (25.4)
Smoking status Never Former Current	159 (60.2) 91 (34.5) 14 (13.3)
Vigorous physical activity (hours per week)^b^	4.2 ± 5.0
College degree^c^	153 (58.2)

SD, standard deviation. ^a^Body mass index data was missing for two subjects. ^b^Physically vigorous activities that cause large increases in heart rate or breathing, such as sports activities, climbing stairs, heavy gardening, or lifting/carrying heavy objects. ^c^Education data were missing for one subject.

**Table 2:** Distribution of serum phthalates, parabens, and phenols in study participants (*N *= 264), Wisconsin Breast Density Study, 2008 to 2009

	Limit of detection	No. (%) with detectable levels	Median detectable value^a^	Maximum detected value
Mono-ethyl phthalate (ng/ml)	0.11	37 (14.1)	3.77	55.8
Mono-butyl phthalate (ng/ml)	1.00	5 (1.9)	NA^b^	136
Mono-benzyl phthalate (ng/ml)	0.10	1 (0.4)	NA^b^	0.2
Propyl paraben (ng/ml)	0.07	175 (66.5)	0.46	630.0
Butyl paraben (ng/ml)	0.02	143 (54.2)	0.13	2.26
Octylphenol (ng/ml)	0.25	35 (13.3)	1.78	58.2
Nonylphenol (ng/ml)	0.06	109 (41.3)	16.8	725.0
Bisphenol A (ng/ml)	0.24	69 (26.3)	0.55	8.77

^a^Refers to the median of serum levels among samples with detectable values (that is, excluding nondetectable samples). ^b^Not available; summary statistics were not calculated because of insufficient numbers of subjects with detectable levels.

Percentage breast density was square-root transformed to improve the normality of the data. Multivariable linear regression was used to assess the association between each xenoestrogen blood measure and the square root of percentage breast density, while adjusting for age, body mass index (BMI), and other variables that previously were shown to be associated with density in this study population: parity, family history of breast cancer, vigorous physical activity, and pack-years of smoking [27]. Visual inspection of the data revealed two outlier values of mono-ethyl phthalate (92 ng/ml and 130 ng/ml) and two outlier values of bisphenol A (10.7 ng/ml and 14.5 ng/ml), which were excluded from all analyses. Sensitivity analyses revealed that exclusion of these outliers did not influence the interpretation of the results. To illustrate the magnitude of the difference in percentage breast density according to xenoestrogen levels, separate models included each xenoestrogen serum level categorized as nondetectable, below the median of detectable values, and above the median of detectable values. Adjusted least-squares mean levels of square-root percentage density were calculated according to these categorized groups and reverse transformed for display purposes. Trend tests across categorized groups were conducted by including the serum-level category as an ordinal term in the regression models. We hypothesized that xenoestrogen chemicals may influence breast tissue more strongly in a low endogenous hormone environment. Tests for modification of the relation between xenoestrogens and percentage breast density by serum estradiol and BMI were conducted by including continuous cross-product interaction terms in the regression models.

Finally, all analyses were repeated by using the square root of dense area (rather than percentage density) as the outcome of interest.

## Results

Table 1 summarizes the characteristics of the study subjects. The mean age of participants was 60.6 years (standard deviation, 4.4 years). About 31% of participants were overweight, and 37% were obese. In general, the study population was highly educated (80.7% had attended at least some college) and reported low smoking rates (60.2% had never smoked).

The distributions of the measured serum phthalates, parabens, and phenols are described in Table 2. Propyl paraben and butyl paraben were detected in more than half of the study samples. Mono-ethyl phthalate, octylphenol, nonylphenol, and BPA were detected in 13% to 41% of samples. Mono-butyl phthalate and mono-benzyl phthalate were detected in very few samples (1.9% and 0.4%, respectively) and were not considered in further analyses. Table 3 presents the Spearman correlation coefficients between each of the xenoestrogens and subject age, BMI, and serum estradiol. A number of xenoestrogens exhibited modest correlations with each other, the strongest of which was between propyl and butyl paraben (*r *= 0.36). Moderate correlation was found between nonylphenol and estradiol (*r *= 0.20). No statistically significant correlations were observed between xenoestrogen values and subject age or BMI.

**Table 3:** Spearman correlation coefficients between serum xenoestrogens and other subject characteristics (*N *= 264), Wisconsin Breast Density Study, 2008 to 2009

	Spearman correlation coefficient (*P *value)								
							
	Mono-ethyl phthalate	Propyl paraben	Butyl paraben	Octylphenol	Nonylphenol	Bisphenol A	Age	BMI	Estradiol
Mono-ethyl phthalate	1.00	0.16 (0.01)	-0.05 (0.43)	-0.05 (0.43)	-0.05 (0.41)	-0.07 (0.23)	-0.01 (0.89)	-0.01 (0.84)	-0.06 (0.31)
Propyl paraben		1.00	0.36 (<0.01)	0.06 (0.37)	-0.04 (0.55)	-0.01 (0.88)	-0.04 (0.52)	-0.08 (0.20)	0.002 (0.97)
Butyl paraben			1.00	0.12 (0.05)	0.11 (0.09)	0.03 (0.65)	-0.10 (0.10)	-0.05 (0.43)	0.09 (0.16)
Octylphenol				1.00	0.11 (0.07)	0.11 (0.06)	-0.11 (0.06)	-0.02 (0.72)	0.04 (0.55)
Nonylphenol					1.00	0.15 (0.02)	-0.05 (0.38)	0.05 (0.38)	0.20 (<0.01)
Bisphenol A						1.00	0.03 (0.67)	-0.09 (0.16)	-0.04 (0.50)

BMI, body mass index.

In the multivariable-adjusted linear regression models, the square root of percentage density was positively associated with mono-ethyl phthalate (β = 0.034; *P *= 0.01) and BPA (β = 0.191; *P *= 0.01). No evidence was noted for an association between percentage breast density and propyl paraben, butyl paraben, octylphenol, or nonylphenol serum levels when treated as continuous variables (all *P *> 0.10).

Results from the regression models using categorized serum xenoestrogen levels are displayed in Table 4. Mean percentage density was 12.6% among women with nondetectable BPA levels, 13.7% among women with detectable levels below the median (<0.55 ng/ml), and 17.6% among women with detectable levels above the median (*P*_trend _= 0.01). Percentage breast density was also elevated (18.2%) among women with serum mono-ethyl phthalate above the median detected level, but the trend was of borderline statistical significance (*P*_trend _= 0.07). No evidence was found for a trend in percentage breast density with increasing categories of propyl paraben, butyl paraben, octylphenol, or nonylphenol levels.

**Table 4:** The association of serum phthalates, parabens, and phenols with mammographic breast density (*N *= 264), Wisconsin Breast Density Study, 2008 to 2009

	Mean percentage density (95% confidence interval)^a,b^			
		
Chemical	Nondetected	Below median^d^	Above median^d^	*P*_trend_^c^
				
	*n *= 225	*n *= 19	*n *= 18	
Mono-ethyl phthalate	13.1 (11.9, 14.3)	13.0 (9.1, 17.7)	18.2 (13.4, 23.7)	0.07
				
	*n *= 88	*n *= 88	*n *= 87	
Propyl paraben	12.7 (10.9, 14.8)	13.3 (11.4, 15.4)	13.9 (12.0, 16.1)	0.41
				
	*n *= 121	*n *= 72	*n *= 71	
Butyl paraben	14.4 (12.7, 16.3)	12.4 (10.4, 14.7)	12.4 (10.4, 14.7)	0.13
				
	*n *= 228	*n *= 19	*n *= 16	
Octylphenol	13.5 (12.3, 14.7)	11.3 (7.6, 15.6)	14.5 (9.9, 19.9)	0.93
				
	*n *= 155	*n *= 55	*n *= 54	
Nonylphenol	14.1 (12.6, 15.6)	12.1 (9.8, 14.7)	12.5 (10.1, 15.1)	0.21
				
	*n *= 193	*n *= 35	*n *= 34	
Bisphenol A	12.6 (11.4, 14.0)	13.7 (10.7, 17.1)	17.6 (14.1, 21.5)	0.01
				

SE, standard error. ^a^Adjusted for age, body mass index, parity, family history of breast cancer, vigorous physical activity, and smoking. ^b^Mean percentage density displayed is reverse transformed from model of square root density. ^c^*P*_trend _refers to the test for the ordinal variable representing nondetected, below median detected value, and above median detected value in a separate model. ^d^Refers to the median of detected serum levels (that is, excluding nondetectable samples).

**Figure 1:**
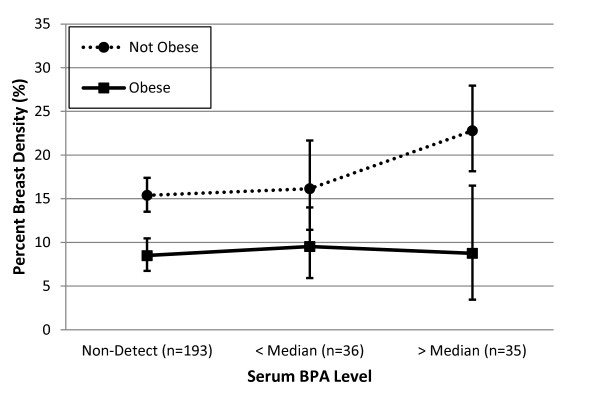
**The association between serum BPA and percent breast density by obesity status, Wisconsin Breast Density Study, 2008-2009**. Percentage breast density shown is reverse transformed from regression model of square root percentage density and adjusted for age, parity, family history of breast cancer, vigorous physical activity, and smoking; error bars indicate 95% confidence limits. The data include 96 obese women and 166 women who were not obese.

We assessed whether the associations of BPA and mono-ethyl phthalate with percentage breast density varied according to measures of the endogenous hormone environment, including BMI and serum estradiol. No evidence was seen for an interaction between mono-ethyl phthalate and BMI or estradiol. The association between BPA and percentage breast density varied according to BMI (*P*_interaction _= 0.03). BPA levels were positively associated with percentage density only among women who were not obese (Figure 1). However, the association of BPA with percentage breast density did not appear to be modified by serum estradiol level (*P*_interaction _= 0.58).

In general, similar results were obtained when evaluating the relation between each xenoestrogen chemical and dense breast area (rather than percentage density). Multivariable-adjusted regression revealed positive associations between dense area and mono-ethyl phthalate (*P*_trend _= 0.03) and BPA (*P*_trend _= 0.08). Effect modification of the association between BPA and dense area by body mass index was not statistically significant (*P*_interaction _= 0.31), but a similar pattern was observed in which the elevation of dense area with high BPA levels was largely restricted to women who were not obese (data not shown).

## Discussion

This study provides the first evidence that mammographic breast density varies according to circulating serum levels of xenoestrogens in postmenopausal women. We found that serum levels of BPA and mono-ethyl phthalate were associated with elevated percentage breast density independent of BMI and other covariates. For both chemicals, percentage breast density was elevated by about 5 percentage points among women with serum levels above the median detected value compared with women with undetectable levels. Mammographic breast density is known to be one of the strongest risk factors for breast cancer [21,22]. Previous studies suggest that a 5-percentage-point difference in percentage density corresponds to an approximately 5% to 10% increase in breast cancer risk [33,34]. For comparison, an absolute difference of 5 percentage points in percentage breast density is similar to the average increase in percentage density observed after 1 year of estrogen-plus-progestin postmenopausal hormone use [24,35], which is a known breast cancer risk factor.

To our knowledge, no previous studies have evaluated mammographic breast density in relation to biological measures of phthalate, paraben, or phenol exposures. We are aware of only one study examining the relation between these chemicals and breast cancer risk in humans. A case-control study examined breast cancer risk in relation to phthalates in urine samples from Mexican women [20]. Women with urinary mono-ethyl phthalate levels in the highest tertile were more than twice as likely to have breast cancer as were women in the lowest tertile (odds ratio, 2.2; 95% CI, 1.33 to 3.63). Our finding of elevated percentage breast density among women with high circulating serum levels of mono-ethyl phthalate is consistent with this finding.

Humans are generally exposed to phthalates as diesters in consumer products. The metabolism of these diesters is rapid, with elimination half-lives generally less than 24 hours [36]. Excretion of phthalate metabolites occurs primarily via urine [1]. Mono-ethyl phthalate is the primary metabolite of diethyl phthalate. Products that may contain diethyl phthalate include perfumes, deodorants, soaps, shampoos, cosmetics, and lotions [1].

BPA is widely used in plastics and cans for food packaging, and BPA exposure is considered to occur predominantly through food [3]. After ingestion, BPA is metabolized through glucuronidation, with acute-exposure studies suggesting an elimination half-life in the body of about 4 to 6 hours [3]. However, a recent NHANES study suggested that either substantial nonfood sources of exposure exist, or substantial accumulation of BPA occurs in body compartments with long elimination times [37]. Despite its short half-life in the body, BPA appears to be present in adipose tissue in its lipophilic unconjugated forms [38]. Release of free BPA from adipose tissue may represent a source of continuous exposure for target organs [6].

The mechanisms by which mono-ethyl phthalate or BPA exposure could influence mammographic breast density are unclear. *In vitro *and *in vivo *assays indicate that phthalates and BPA have estrogenic activity [8,9]. The serum concentration of these chemicals in our study population was about 100 to 1,000 times higher than that of estradiol; however, the estrogenic potency of mono-ethyl phthalate and BPA is believed to be 10,000 to 1 million times less than that of estradiol. *In vitro *experiments and human studies provide inconsistent evidence for mutagenicity [16,39], and animal studies have revealed limited evidence for impacts on the mammary gland in adult animals [1,40]. However, evidence suggests that the offspring of rats exposed to BPA during pregnancy exhibit altered mammary gland architecture during puberty and adulthood, including an increased number of hyperplastic mammary ducts, increased stromal nuclear density, and increased terminal end-bud density [41,42]. Additionally, a recent study reported that urinary BPA levels were associated with upregulated estrogen receptor and estrogen-related receptor expression among adult men [43]. Recent studies have also revealed that environmentally relevant doses of BPA can influence adiponectin production in human adipose tissue, which could influence insulin sensitivity and tissue inflammation [44].

We explored potential interactions between the xenoestrogen exposures and the internal hormone environment. We had hypothesized that the role of xenoestrogens may be attenuated in women with higher endogenous hormone levels. The association between BPA and percentage breast density was limited to women who were not obese, which was consistent with our hypothesis, because obese women have higher endogenous hormone levels [45]. However, when we directly examined effect modification by endogenous estradiol levels, we found no evidence for an interaction. The interpretation of these findings is somewhat unclear, but they suggest that the role of obesity in effect modification may be independent of a sex hormone pathway. Notably, endogenous estrogens do not appear to be strongly associated with mammographic breast density [27], whereas obesity has a strong inverse association with mammographic breast density that is independent of hormone levels. The strong predisposition of obese women to have low mammographic density may limit any potential effect of BPA within this subgroup. Alternatively, the lack of effect modification by estradiol may be due to the small sample size or the limited range of estradiol levels observed in this sample of postmenopausal women. Given the limited power and precision for estimating effect modification, these findings require replication and should be interpreted with caution. The results do suggest that future studies should test effect modification by BMI, and if possible, by endogenous hormones that affect breast cancer risk, because the role of environmental exposures may vary across these subgroups.

The primary limitation of our study is the use of a single serum measurement to assess exposure levels. As described earlier, the metabolism and excretion of phthalates, parabens, and phenols is rapid and efficient. Although the pharmacokinetics of mono-ethyl phthalate and BPA metabolism are not completely understood, it is clear that these chemicals have short elimination half-lives. Thus, serum measurements largely reflect exposures within the past 24 hours. The correlation of one serum measurement with exposure history is unknown for these chemicals, and we are unaware of prior studies that have examined multiple serum measurements of BPA or phthalates in individuals over time. Prior studies have most often used urine samples to evaluate exposure levels in population studies, because phthalate and BPA concentrations are about 20 to 100 times higher in urine than in blood [39,40]. Some evidence indicates low to moderate correlation between urinary BPA and phthalate measures taken a month or more apart, with observed intraclass correlation coefficients of approximately 0.1 to 0.3 for BPA and 0.3 to 0.5 for mono-ethyl phthalate [46-50]. Misclassification of subject exposure levels would tend to bias our results toward a null association and would thus not appear to explain the observed positive associations. It is also possible, however, that the associations between circulating levels of monoethyl phthalate and BPA and percentage breast density may be due to confounding by an unmeasured factor that influences both xenoestrogen metabolism and mammographic breast density.

Despite the limitations with xenoestrogen serum measurements, they were previously shown to demonstrate associations with polycystic ovarian syndrome, obesity, and recurrent miscarriage [14,41]. One potential advantage of serum measurements is that they may more directly assess circulating concentrations that reflect the biologically relevant exposure of the target organs [6].

Further investigation with more comprehensive exposure measurements and longitudinal study designs will be necessary to confirm and further examine the associations observed in our study. Studies are also needed in more diverse populations, as our study subjects were predominantly non-Hispanic whites (97% of our study subjects), highly educated, limited in age range, and had a high prevalence of family history of breast cancer. Although these attributes are consistent with the source population of women undergoing screening mammography in our recruitment centers, future studies would benefit from inclusion of more-diverse study subjects with a wider range of exposure levels.

## Conclusions

The results of this study indicate that serum levels of mono-ethyl phthalate and BPA are cross-sectionally associated with elevated mammographic breast density in postmenopausal women. Given the widespread exposure of the population to these chemicals and the strong association between mammographic breast density and breast cancer risk, further research is warranted to examine the potential role of these chemicals in breast carcinogenesis in humans.

For mono-ethyl phthalate, the consistency between our findings and those of a previous case-control study of breast cancer risk are particularly striking. The results observed here must be confirmed in larger study populations. Future studies evaluating these exposures in relation to mammographic breast density or breast cancer risk should seek to use longitudinal study designs, multiple exposure assessments, and diverse study populations.

## Abbreviations

BPA: bisphenol A; BMI: body mass index; CI: confidence interval; NHANES: National Health and Nutrition Examination Survey; SD: standard deviation; SE: standard error.

## Competing interests

The authors declare that they have no competing interests.

## Authors' contributions

BLS participated in the conception and design of the study, the acquisition of data, the statistical analyses, and drafted the manuscript. AT participated in the conception and design of the study, the acquisition of data, and critical revision of the manuscript. CJH participated in the design of the study, the acquisition of data, and critical revision of the manuscript. JW participated in the statistical analyses and critical revision of the manuscript. JDCH participated in the design of the study and critical revision of the manuscript. JMH participated in the design of the study and the statistical analyses. DSMB participated in the design of the study, the acquisition of data, and critical revision of the manuscript. EJAB participated in the design of the study, the acquisition of data, and critical revision of the manuscript. GSS participated in the design of the study, the acquisition of data, and critical revision of the manuscript. ESB participated in the design of the study, the acquisition of data, and critical revision of the manuscript. All authors read and approved the final manuscript.
